# Exploring how complex multiple-choice questions could contribute to inequity in introductory physics

**DOI:** 10.1371/journal.pone.0323813

**Published:** 2025-05-30

**Authors:** Nicholas T. Young, Mark Mills, Rebecca L. Matz, Eric F. Bell, Caitlin Hayward

**Affiliations:** 1 Department of Physics and Astronomy, University of Georgia, Athens, Georgia, United States of America; 2 Center for Academic Innovation, University of Michigan, Ann Arbor, Michigan, United States of America; 3 Department of Astronomy, University of Michigan, Ann Arbor, Michigan, United States of America; Christian Medical College Vellore, INDIA

## Abstract

**Introduction:**

High-stakes exams significantly impact introductory physics students’ final grades and have been shown to be inequitable, often to the detriment of students identifying with groups historically marginalized in physics. Certain types of exam questions may contribute more than other types to the observed equity gaps.

**Objective:**

The primary objective of this study was to determine whether complex multiple-choice (CMC) questions may be a potential cause of inequity.

**Methods:**

We used four years of data from Problem Roulette, an online, not-for-credit exam preparation program, to address our objective. This data set included 951 Physics II (Electricity and Magnetism) questions, each of which we categorized as CMC or non-CMC. We then compared student performance on each question type and created a multi-level logistic regression model to control individual student and question differences.

**Results:**

Students performed 7.9 percentage points worse on CMC questions than they did on non-CMC questions. We find minimal additional performance differences based on student performance in the course. The results from mixed-effects models suggest that CMC questions may be contributing to the observed equity gaps, especially for male and female students, though more evidence is needed.

**Conclusion:**

We found CMC questions are more difficult for everyone. Future research should examine the source of this difficulty and whether that source is functionally related to learning and assessment. Our data does not support using CMC questions instead of non-CMC questions as a way to differentiate top-performing students from everyone else.

## Introduction

High-stakes exams make up a substantial portion of a student’s final grade in introductory physics courses [1,2]. These exams generally consist of forced-choice questions. Prior work has found introductory physics exams are inequitable. For example, men tend to outperform women [3,4], a trend seen more generally across STEM courses [5,6].

Given that exam grades are inequitable in physics, it is possible that individual multiple-choice questions on exams are also inequitable and in sum, produce some part of the overall grade inequities observed on exams. For example, various physics studies have noted that the presentation and context of a question, independent of the underlying physics content, can lead to gendered performance differences [7–9], though these studies have typically focused on the wording, rather than the layout of the question.

**Fig 1:**
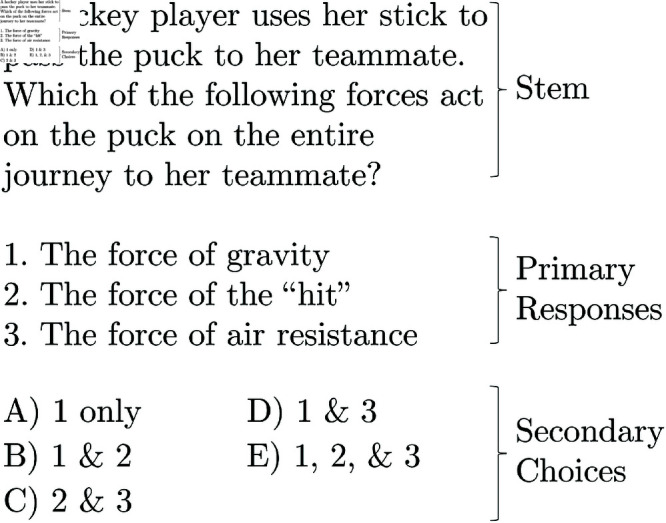
A sample CMC question. The stem, primary responses, and second choices are labeled.

In this study, we take a step toward addressing this issue by examining a specific type of multiple-choice question, the complex multiple-choice (CMC) question, which has seen limited study within physics education. A CMC question is one in which the student is given multiple possible responses to a question and must select the answer choice including all correct responses (see Fig 1 for an example). CMCs are assumed to require and measure higher-order thinking skills [10], but at the same time may add unnecessary cognitive load unrelated to the physics content and potentially benefit some students for reasons unrelated to their physics knowledge.

In this paper, we ask if CMC questions could exacerbate inequity in introductory physics courses or equivalently, if CMC questions could disproportionately benefit some students compared to others. We break this overarching question into five sub-questions:

1. How does students’ performance on CMC questions compare to their performance on non-CMC questions in introductory physics?

Because the format of CMC questions allows multiple correct primary responses, CMC questions often test concepts rather than calculations. We assume this to be the case for all CMC questions in this study. As prior work has found that students often perform better on “plug-and-chug” calculation questions compared to conceptual questions [11], it is possible that any performance gaps found could be a result of that. Therefore, to clarify the answer to question one, we additionally ask:

2 How does the type of knowledge students need to answer a question (conceptual vs. “plug-and-chug”) account for performance differences between CMC questions and non-CMC questions?

Finally, to determine how CMC questions may exacerbate inequity in introductory physics courses, we ask three additional questions:

3 What is the relationship between students’ prior preparation and their ability to answer CMC questions correctly?
4 How well can a CMC question differentiate between students who go on to earn a high versus low grade in their physics course?
5 How might CMC questions affect performance gaps between students of various demographic groups?

The remainder of the paper is organized as follows. First, we provide an overview of existing research on CMC questions, both in general and in physics specifically. Second, we describe how we obtained the data from our Problem Roulette database and how we created the groups used in our analysis. Next, we show the results of descriptive statistics and regression models, finding that CMC questions are harder for students. We then compare the results obtained by the two analytical methods. Finally, we consider how our data source limits the generalizability of the results and suggest directions for future work.

## Background

### Complex multiple-choice questions

The CMC question format is one in which a question is presented with a stem, a set of primary responses, and a set of secondary choices that contain various combinations of the primary responses [12]. Multiple-choice questions that do not follow this format, even if they require a student to select multiple answer choices, are not considered CMC questions for the purposes of this paper. Further, in this paper, we do not differentiate between CMC questions and type-K questions, which are a specific subtype of CMC questions with four primary responses and five of the possible sixteen responses displayed as secondary choices [12].

CMC questions are often used instead of more traditional multiple-choice questions due to the assumed benefits they provide. For example, the higher complexity of the question is assumed to enable assessment of greater complexity in thinking [13]. Given that traditional multiple-choice questions may hinder critical thinking skills in introductory science courses [14], CMC questions could provide a remedy while retaining the benefits of multiple-choice questions such as being easy to grade and implement in large-enrollment courses.

In addition, the CMC format can identify when students believe more than one response is true. A study in biology found that as many as half of students who correctly answered a traditional multiple-choice question would have also (incorrectly) selected another answer choice if given the option [15]. While that study was done with multiple-true-false questions instead of CMC questions, it suggests that traditional multiple-choice questions might not be able to capture the partial understandings students might hold. Further, the CMC format can also be useful for assessing knowledge in cases when there is more than one correct answer.

Despite the proposed benefits of this type of question, CMC questions also have disadvantages relative to traditional multiple-choice questions. First, studies in educational assessment, mathematics, and medicine have found that CMCs are harder for students than traditional multiple-choice questions [12,16–18], though the effect may depend on whether the question requires the recall of memorized information or higher-order thinking skills [19]. As a result, some have claimed that the reason for the difference in performance could be because CMCs are measuring more than just knowledge such as non-cognitive traits [17]. For example, after reformatting CMC questions as multiple-true-false questions, one study found that student performance improved [17].

Second, CMCs may inadvertently “clue” students to the correct answer [12] because not all combinations of primary responses can be present in the secondary choices if there are five or fewer answer choices (a common practice on exams). For example, in Fig 1, knowing that response 2 is incorrect eliminates answers B, C, and E. Of the remaining options, both have response 1. Therefore, a student is potentially able to get the question correct only by knowing whether responses 2 and 3 are true or false. This clueing could then help less knowledgeable students perform better on CMC questions than they would on comparable traditional multiple-choice questions [12].

Third, CMC questions might be inequitable, though the evidence is limited. One study of undergraduates in a teaching certification program found that some, but not all CMC questions in their sample showed performance gaps between male and female students [20].

Finally, there are test construction concerns about CMC questions. CMC questions have been shown to have lower reliability than traditional multiple-choice questions or multiple-true-false questions [10]. In addition, CMC questions can be more difficult to construct [10], although others disagree and believe they are easier to construct because they require fewer distractor answers than traditional questions [21].

Given the number of concerns about CMC questions, many have recommended against using the format [12,13,22]. Yet, recent work suggests that this format is still in use [23–26].

### Complex multiple-choice questions in physics

There has been limited study of the CMC question format in physics. One study [27] found that students performed better on CMC questions than on equivalent versions in the multiple-true-false format with approximately one-third of students answering the multiple-true-false questions in a way that was not represented among the secondary choices in the corresponding CMC format. The study concluded that the CMC format may overestimate student knowledge.

Despite the limited direct study of CMC questions in physics, there have been many indirect studies due to their appearance on many concept inventories including the Test of Understanding of Graphs in Kinematics (items 12 and 19) [28], the Force Concept Inventory (items 12 and 22 on the original 1992 version; items 5, 18, 29, and 30 on the 1995 revision) [29], and the Physics Inventory of Quantitative Literacy (item 4) [30]. In the case of the 1995 version of the Force Concept Inventory, various studies have commented on the CMC items, noting that items 5 and 18 were able to distinguish between high- and low-proficiency students [31]; items 5, 18, and 29 are “problematic” [32]; and item 29 is “potentially malfunctioning for currently unknown reasons” [33]. Thinking toward the next generation of concept inventories in physics (e.g., Laverty and Caballero [34]), it is important to consider whether CMC-type questions should be avoided on these types of assessments.

CMC questions in physics have also appeared in studies of in-class interventions [35], of K-12 contexts [36], of teacher knowledge [37], and student learning [26]. Outside peer-reviewed studies and conceptual inventories, CMC questions have further appeared on the American Association of Physics Teachers’ *F* = *ma* exam, the first qualifier for the US Physics Team in the International Physics Olympiad, and on the physics Graduate Record Examination (GRE) which, until recently, was required for most applicants to physics graduate programs [38].

## Methods

### Data collection

Data for this study comes from Problem Roulette users enrolled in a second-semester calculus-based introductory physics course (referred to as Physics II herein) between the Winter 2019 and Winter 2023 semesters (13 semesters total, including summer semesters which enroll significantly fewer students than Fall and Winter offerings). Problem Roulette is a free, optional, not-for-credit, test question practice software offered to all students in introductory physics courses (and other introductory STEM courses) at the University of Michigan [39]. Students have the option to select a broad physics topic or specific exam period (e.g., Exam 1) and then are randomly served practice questions from the system. Students are given as many attempts as they need to answer the question and are told whether they are correct or not after each response. All questions are uploaded by instructors and appeared on exams during previous course iterations. Prior work has found that using this system regularly results in a significant increase in a student’s final grade in introductory physics over and above what might be expected based on their prior academic preparation (i.e., grades in their other college courses and ACT/SAT scores) [39,40]. For the time period in this study, 1685 students answered at least one Physics II question in Problem Roulette, corresponding to 41% of the students enrolled in the course and 39% of students who completed the course during the study period.

Data for this study also came from a simplified version of the university’s student data warehouse intended to provide researchers with straightforward access to student data [41]. Specifically, we had access to students’ course grades, birth sex, race/ethnicity, high school zip code, parental education status, and residency status.

This study was reviewed by the Institutional Review Board at the University of Michigan and determined to be exempt (HUM00158291). The University of Michigan owns the Problem Roulette software and the data used in this study. The data used were not collected specifically for research and, as such, are secondary data in this study. The terms of service users agree to before using Problem Roulette allow the university to perform research related to evaluating the performance of users. In addition, the Family Education Rights and Privacy Act (FERPA) allows student educational records to be used for research without consent if the goal of the research is to improve instruction. Therefore, the typical requirement to obtain active consent from participants was waived and not sought by the researchers.

To protect student privacy, potentially identifying information was stored separately from student responses and grades. Student responses and grades were assigned random numbers via Problem Roulette and the university when creating the data files. A linking file was then used to connect the two data sources and match students in each database. The connection was done via R [42] rather than manually so that no member of the research team could potentially associate a random identifier with potentially identifying information. After students were matched, the potentially identifying information in the linking file was removed from the resulting data set.

### Demographic definitions

Noting that demographic categories are neither natural nor given [43], in this section, we discuss how we defined each demographic group. As the demographic data is collected by the university and not directly by us as researchers, we had no control over the options students were able to select from and acknowledge that they are not the only axes on which privilege and oppression act in physics. The five demographic categories were sex, race/ethnicity, socioeconomic status, parental education, and international status. We provide the definitions and criteria for creating groups in each of these categories below and then state why we choose to include these demographic variables.

- **Sex**: Sex was recorded as a binary variable (male or female) even though sex is not binary [44] and gender is actually the construct of greater interest.
- **Race/ethnicity**: Students were able to select any of the following five options: Asian, Black, Hispanic, Native American, and white. Any student who selected more than one option was marked as Multiracial. Due to the limited numbers of Black, Hispanic, Multiracial, and Native American students in our study population, we grouped these students into one category and we grouped Asian and white students into a second category. Given that the underrepresented minority label is considered racist and harmful [45–47], we instead refer to these groups as “B/H/M/N” and “A/w”, using the first letter of each race and ethnicity recorded by the university, following the capitalization used by Robertson *et al*. in a recent physics education article[48]. We acknowledge that such groupings ignore the unique situations of each group and further mask the experiences of individuals [45,49,50].
- **Socioeconomic status**: We defined a student as low-income with a binary variable where a student was considered low-income if the median annual household income of the student’s high school’s zip code was less than $60,000. We determined median annual household income by zip code using the 2020 American Community Survey 5-Year Estimates. We used this metric rather than the student’s self-reported estimated family income—another socioeconomic metric available to us—due to a large amount (more than 20%) of missing data and a lack of access to data about which students received Pell Grants, a common choice for denoting low-income students in education research. We also use this community-based definition of low income rather than an individually-based definition to acknowledge that educational resources students have access to are not solely based on their family but also their community. Therefore, a low-income student based on family income may have had access to different opportunities if they attend a well-resourced school compared to a low-income student based on family income who attends a less-resourced school. Students without a zip code listed (such as international students) were conservatively coded as not low-income.
- **Parental education**: We defined first-generation status as a binary variable, where any student with neither parent earning at least a bachelor’s degree was marked as a first-generation student. Students could select their parents’ education level as one of eleven levels. We grouped elementary school only, less than high school, high school diploma, some college, Associate’s degree, and nursing diploma into the first generation category, and we grouped Bachelor’s degree, Master’s degree, Professional Doctorate, Doctorate, and Post Doctorate into the continuing generation category.
- **International status**: International student status was based on whether the university considers the student to be international. The university from which the data come classifies a student as international if the student has a citizenship status of Non-Resident Alien or Alien Under Tax Treaty.

Our reasons for including these variables are as follows. We include sex and race/ethnicity as these are common demographic variables studied in physics education when examining equity issues. We include socioeconomic status and first-generation status as we assume that middle- and upper-class students and students with college-educated parents may have a greater understanding of the hidden curriculum of college, which may include how to approach and answer exam questions presented in non-standard formats such as CMC questions. Finally, we include international status under the assumption that most international students do not speak English as their first language, and the additional cognitive load of CMC questions could be especially impactful for students who are not native English speakers.

### Analysis

**Fig 2:**
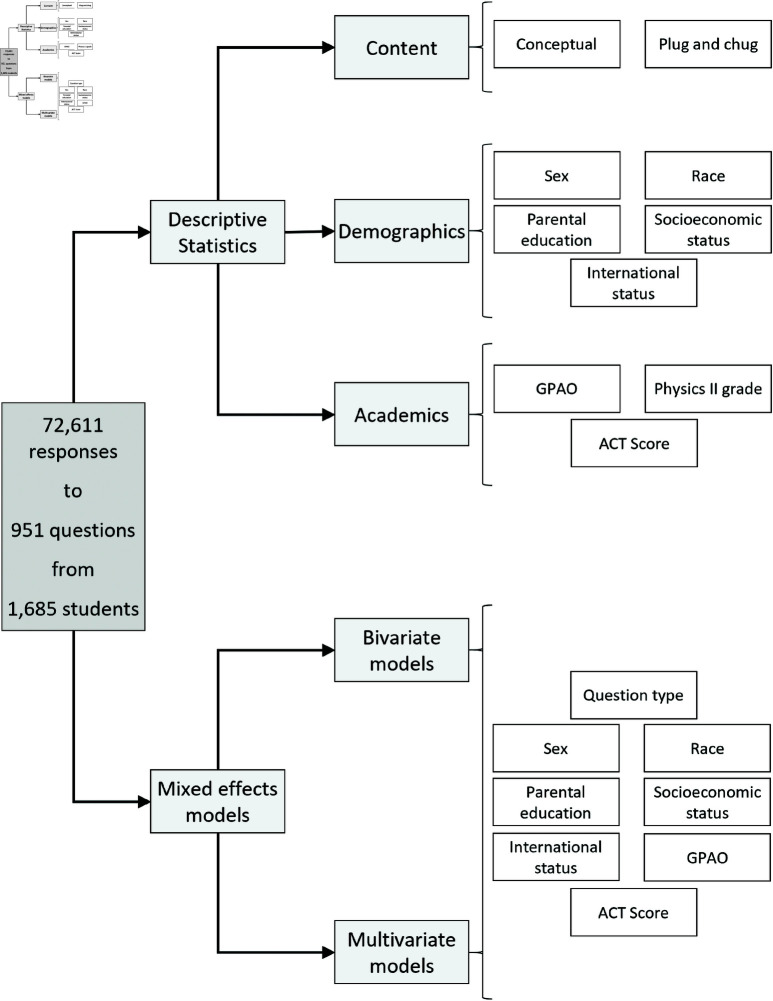
Summary of analysis methods used in this study. Light grey boxes represent the categories of analysis while white boxes represent the variables included at each stage of the analysis.

Here, we describe our procedures for analyzing the data. The various analyses are summarized in Fig 2 and are described below.

#### Data cleaning and handling.

We first matched all responses to Physics II questions in Problem Roulette to the university’s enrollment records and removed responses from students who were not enrolled in the course and any responses that could not be matched to an enrollment record. We also removed responses that were blank and hence not marked as correct or incorrect by the system, which happens when the user is delivered a question and then exits the platform without answering it (presumably because they are done studying for the time being) as well as any question that a student chose to skip. Finally, we removed responses that were marked as “retry” so that our data set only contained the student’s first response to each question, excluding any responses provided after students received feedback and mimicking more typical testing environments in which students respond to each test item only once. We explicitly note this does not make the setting equivalent to that of a testing environment and that the data are still from a low-stakes, not-for-credit environment.

**Fig 3:**
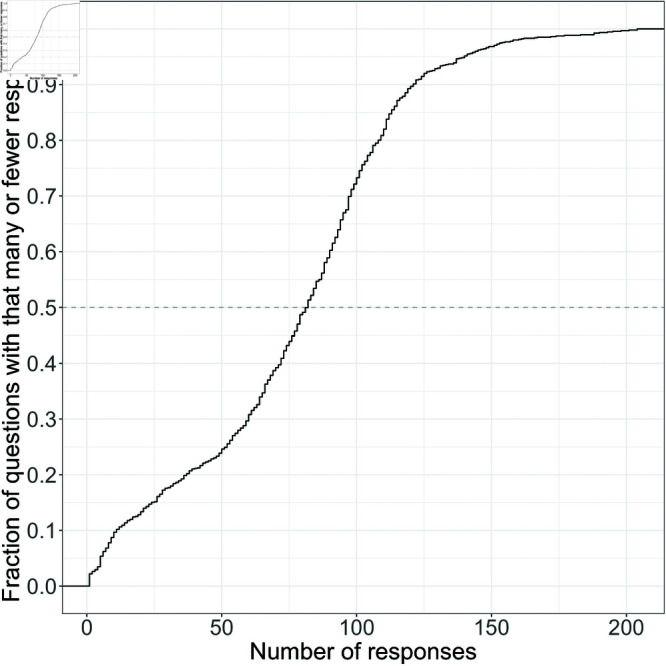
Cumulative distribution of responses to each question in Problem Roulette for Physics II. The dotted line corresponds to the median number of responses, which is 82 for this data set.

We were left with 72,611 responses to 951 questions from 1,685 students, where students and questions were crossed given that each student could theoretically respond to each question. Because questions are served randomly to students, questions have different numbers of responses. In our data set, the most encountered question had 204 responses, the least encountered question had 1 response and the median number of responses was 82. Random effects modeling was used to account for this imbalance [51,52] and is described later in this section. The cumulative distribution of responses is shown in Fig 3.

#### Descriptive statistics.

To address our research questions, we then identified the CMC questions. We searched each question response for one of one hundred strings of text (listed in S2 Appendix) that could be indicative of a CMC question such as “1 and 2”, “both b and d”, or “I, II, and III”. Each question that included an answer pattern matching this list was then manually reviewed to see if it was in fact a CMC question according to our definition presented in the background. For example, a question that asked students to rank the brightness of light bulbs in a circuit might contain the string “I, II, and III” and hence be errantly flagged as a CMC question. From this exercise, we identified 44 CMC questions and 907 non-CMC questions in our data set. For both types of questions, we summed the total number of correct first responses and divided by the number of first responses to get overall accuracy rates.

To determine how much of the performance difference between CMC questions and non-CMC questions could be explained by the differences in the type of knowledge students would need to answer the question, we sampled the non-CMC questions to estimate the percentage of non-CMC questions that were conceptual versus “plug-and-chug”. If performance on CMC questions and conceptual non-CMC questions were similar, it would suggest that the nature of the question (conceptual instead of “plug-and-chug”) rather than the format of the question were responsible for the increased difficulty. However, if performance on CMC questions and conceptual non-CMC questions were different, it would suggest that either the format of the question or the knowledge being assessed were the cause of the difference.

To do so, we categorized the first fifty questions in the Problem Roulette database as conceptual or “plug-and-chug”. If correctly answering the question required a numeric or symbolic calculation more than just a multiplicative factor (e.g., a proportional reasoning question), we marked the question as “plug-and-chug” and otherwise conceptual. Examples of each type of question are included in the supplemental material (S3 Appendix). Afterward, we randomly sampled the remaining questions in the database until we had categorized 15% of the non-CMC questions (136 questions total). In doing so, we found that 37% of our sampled non-CMC questions were conceptual while the remaining 63% were “plug-and-chug”. All of the CMC questions were conceptual.

To account for prior preparation, we used a metric called grade point average in other courses (GPAO) as well as standardized test scores. GPAO is calculated in relation to a specific course and is generally calculated as the student’s grade point average in all other courses the student has taken up to and including the semester the student took the specific course [53]. For example, the GPAO for a student who enrolled in Fall 2019 and took Physics II in Winter 2021 would be their grade point average of all the courses they took between Fall 2019 and Winter 2021 inclusive except for Physics II. GPAO is therefore a measure of how well a student has performed relatively in their courses at their institution and has been found to be more predictive of a course grade than a student’s high school GPA or their SAT or ACT scores [1,54].

Students at the university were able to submit either ACT or SAT scores and thus, both are not recorded for most students. If a student only submitted SAT scores, we used ACT concordance tables to convert from the SAT to ACT score. We chose this conversion because SAT scores map uniquely to an ACT score while an ACT score maps to a range of SAT scores. If the student had both scores, the existing ACT score was used and the SAT score was not converted. Overall, only 58 (3.5%) students had neither an ACT nor SAT score recorded in the student data warehouse.

We then split students into a high-performing group if their GPAO was at least 3.7 (an average grade of A- or better) and otherwise into a lower-performing group and compared their overall accuracy rates on CMC and non-CMC questions. For the purpose of referring to the grades students earned in general (via GPAO), we use “A” to refer to any A-level (A-, A, A+). We discretized this continuous variable because we are interested in whether CMC questions disproportionately help high-performing students. For reference, grades at the university are based on a standard 4.0 scale (A is 4.0, A- is 3.7, B+ is 3.3, B is 3.0, etc.)

Likewise, to understand how CMC questions could differentiate students who would earn a high versus low grade in Physics II, we also categorized students as a high grade earner if they received at least an A- in Physics II and a lower grade earner if they earned a B+ or less; roughly 40% of students were classified as high grade earners (S1 Appendix). We base this choice on the observation that earning an “A” is the most common grade and that the percentage of students earning other letter grades drops off steadily after. We note that B-level grades could also be included in the higher grade category. However, in our experience working with faculty, we have noticed that when faculty refer to students who earned a high grade in their course, they are nearly always referring to students who earned “A”s. Further, including B-level grades would result in around 70% of students being classified as high-grade earners.

To understand how CMC questions may affect performance gaps, we compared performance on CMC questions to non-CMC questions for five demographic groups based on data in our student data warehouse. Across all five variables, students with missing data were only excluded from the analysis for the specific demographic variable that they were missing.

To better understand the impact of question style on accuracy, we looked at performance on CMC and non-CMC questions by individual students. We compared each student’s fraction of correct responses to non-CMC and CMC questions. Due to the possibility that the randomly presented questions the student saw might have only included one or two CMC questions and thus resulted in an exaggerated performance on the questions, we required that the student had responded to at least five CMC questions to be included in this portion of the analysis.

#### Mixed effects models.

Finally, to gain further understanding of any effects observed, we ran a series of mixed effects logistic regression models. We adopt an explanatory modeling approach in that we assume that the variables in our model relate to an underlying set of factors that cause the outcome and our goal is to minimize bias to obtain the most accurate description of the underlying theory [55]. We first ran five bivariate regression models, using one of each of the five demographic variables as a binary variable, question type (CMC or non-CMC), and an interaction term as inputs and whether the question was answered correctly (yes or no) as an output. For each of these models, we included the term the student was enrolled, the question ID, and the student as random intercepts to account for possible baseline performance differences based on the term, the individual question, and the individual student. We also included a random slope based on the student and question type because the relationship between performance on CMC and non-CMC questions might not be the same for all students. If there was a differential effect between the demographic or student groups, the interaction term would be statistically significant. We ran another two bivariate models, one with GPAO (as a continuous variable) and one with ACT score, and question type, an interaction term, and the same random intercepts and slopes as before.

To account for how some of the variables may be measuring similar effects, we ran multivariate logistic regression models including subsets of all five demographic variables, GPAO, ACT score, and all interactions between these variables and question type. These multivariate models also included the random slope and intercepts. As the demographic variables along with GPAO, ACT score, and their interactions adds 14 variables to the model and this can make the model difficult to interpret, we performed a model selection procedure to simplify our final model. We began with including only question type in the model and then adding one additional variable (e.g., sex, socioeconomic status, or GPAO) and its interaction with question type to the model, resulting in seven total models. Variables whose interaction with question type were statistically significant in the bivariate models were added first followed by variables that were statistically significant in the bivariate model, and then finally, variables that were not statistically significant.

To compare the models, we then used a likelihood ratio test [56]. In a likelihood test, the likelihood (or log-likelihood) of two models is compared with the null hypothesis that the simpler model (i.e., the one with fewer variables) is a better fit of the data. If the resulting p-value is less than 0.05, there is evidence that the more complex model is a better fit for the data. Using this approach, we are then able to select the model with the fewest terms, balancing the need to explain the underlying data but also be interpretable by researchers. Any data with missing values in any of the variables considered for the models were dropped prior to model construction.

Comparing the results across the bivariate and multivariate models, there are four possible patterns. If a variable has the same relationship with the outcome variable in both the bivariate and multivariate models and it is statistically significant, that variable likely has explanatory power. In contrast, if the variable is not statistically significant, the variable likely does not have explanatory power. If the variable is statistically significant in one of the models but not the other, there are an additional two cases. If the variable was statistically significant in the bivariate model but not the multivariate model, the variable is likely a proxy and likely correlated with another input variable. Alternatively, if the variable is statistically significant in the multivariate model but not the bivariate model (or if the variable is statistically significant in both models but the direction of the effect is different), the variable is a suppressor variable [57]. In this paper, we are especially interested in determining variables with explanatory power and less interested in understanding variables with proxy or suppressor effects. For the purposes of achieving this goal, if a variable is not present in the final selected model, we will consider it a non-significant variable since its inclusion in the model did not result in a model that was better than the model without it.

**Fig 4:**
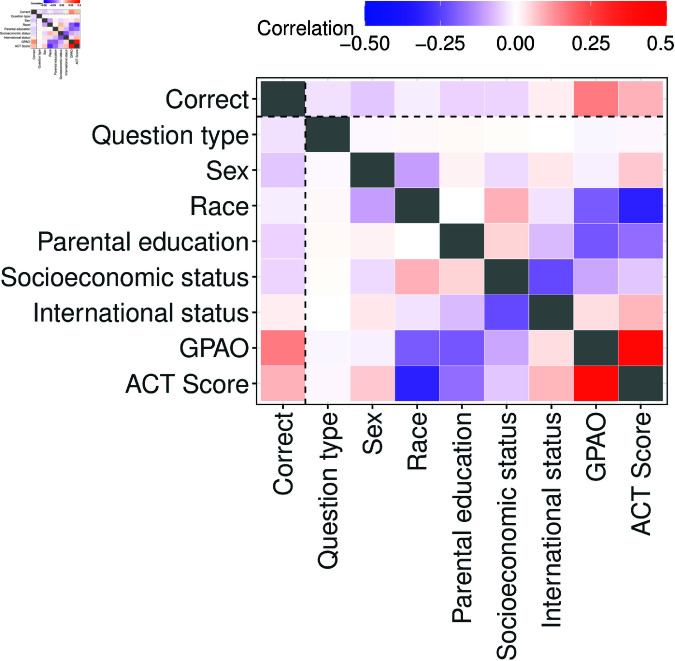
Correlogram of the independent variables in our regression model with each other and the outcome variable, correct. Sex, race, parental education, socioeconomic status, and international status are binary variables with female, B/H/M/N, first generation, low income, and international coded as one.

We performed all analysis in R [42] and used the lme4 package to run the nested models [58]. Following best practices of Kanim and Cid [59] and Young and Caballero [60], the percentage of responses attributed to each variable in our models are shown in Table 1. A correlogram of the data is shown in Fig 4.

**Table 1:** Percent of responses to questions by variables in the regression models by category as well as the amount of missing data.

Variable	Split	Missing
Question type (non-CMC | CMC)	95/5	0
Sex (female | male)	30/70	<1
Race/ethnicity (B/H/M/N | A/w)	13/79	8
Parental education (first-gen | cont.-gen )	10/88	2
Socioeconomic status (low | medium/high)	48/52	<1
Residency (international | domestic)	6/94	<1

Across these models, continuous variables were scaled and centered as appropriate. We centered GPAO and ACT scores based on the average GPAO and ACT among the students in the data set as a GPAO or ACT of zero is highly unlikely to occur in practice. Under this transformation, the intercept corresponds to a student with an average GPAO and average ACT score in this sample (3.49 and 32.75, respectively).

In addition, any cases with missing data were removed. For these models, there were 64,383 usable responses in 950 questions and 1,493 students, where questions and students are crossed, across 13 terms.

## Results

### Descriptive statistics

Examining overall performance, we found that students performed worse on CMC questions compared to non-CMC questions. Students submitted 2,994 responses to CMC questions with an accuracy rate of 44.1% (95% confidence interval: 42.3%, 45.8%) compared to 69,617 responses to non-CMC questions with an accuracy rate of 51.9% (95% CI: 51.5%, 52.3%). That is, the rate at which students correctly answered CMC questions was 7.9 (95% CI: 6.1, 9.7) percentage points lower than that at which they correctly answered non-CMC questions. Despite this apparent increase in difficulty, we found minimal evidence that students skipped CMC questions more than non-CMC questions. There were 149 skipped CMC questions (4.74% of CMC questions served to students) and there were 2,784 skipped non-CMC questions (3.84% of non-CMC questions served to students).

To understand whether this performance difference was due to the format or content of the question (conceptual vs. “plug-and-chug”), we compared student accuracy on a subset of non-CMC questions. We found that 52.5% of responses to “plug-and-chug” non-CMC questions were correct (95% confidence interval: 51.3%, 53.7%) while 48.9% of responses to conceptual non-CMC questions were correct (95% CI: 47.4%, 50.4%).

That is, students performed 4.82 (95% CI 2.50, 7.14) percentage points better on non-CMC conceptual questions than they did on CMC conceptual questions.

**Fig 5:**
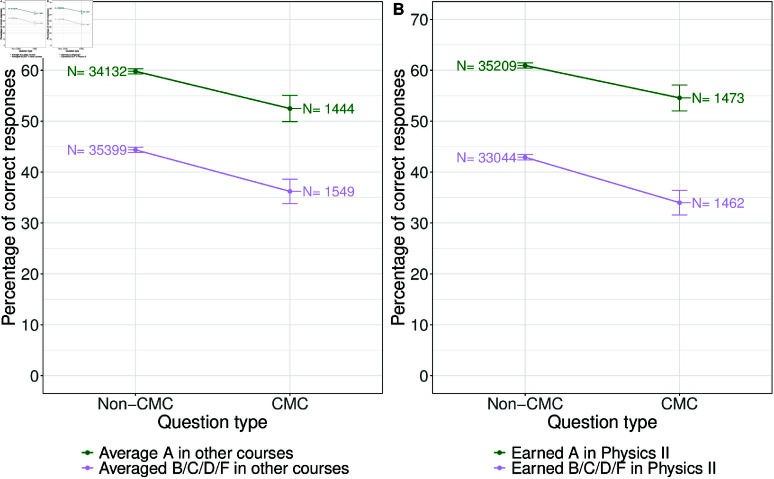
Percent of CMC and non-CMC questions answered correctly based on the average grade students earned in their other classes before or while enrolled in Physics II (panel A) and grade earned in Physics II (panel B). Numbers above each data point are the number of responses from that grade group to that type of question and the error bars are 95% confidence intervals. Both groups of students do equally worse on CMC questions.

The results of taking prior performance and grades earned in Physics II into account when answering CMC and non-CMC questions are shown in Fig 5. Looking at prior performance first (Fig 5A), we notice that regardless of a student’s grades in their other courses, students correctly answered non-CMC questions at a higher rate than CMC questions. However, students who typically earn “A”s in their other courses correctly answered CMC questions at a higher rate than students who typically earned less than “A”s in their other courses. Looking at the discrepancy in performance on the question types between students who earned “A”s in their other courses and those who did not, we find the gap to be about the same size as previously described—around 8 percentage points.

Looking specifically at the grades students earned in Physics II, we find a similar result (Fig 5B). Students who earned “A”s in Physics II correctly responded to non-CMC questions more often than they did CMC questions and correctly answered CMC questions more often than students who didn’t earn “A”s in Physics II correctly responded to non-CMC questions. Looking at the difference in performance between the two question types based on the student’s Physics II grade, we notice that the gap is slightly smaller for students who earned “A”s compared to those who did not (6.4 vs. 8.9 points).

**Fig 6:**
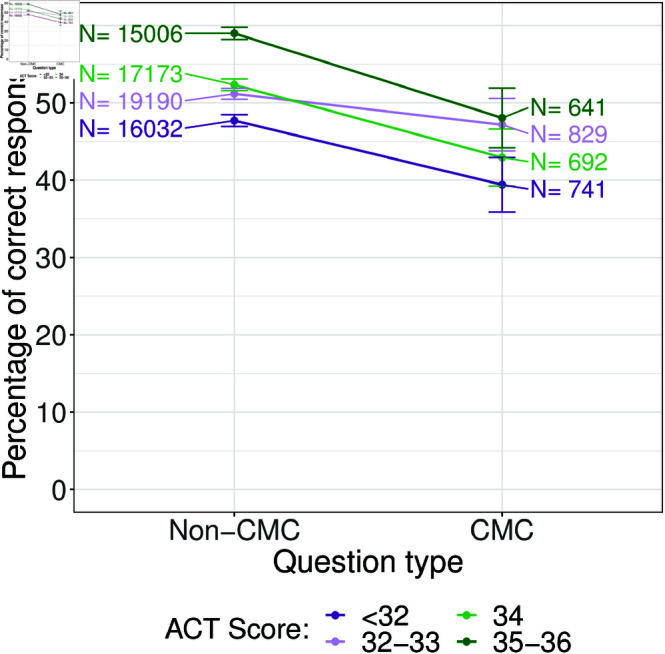
Percent of CMC and non-CMC questions answered correctly based on student’s ACT score. Higher ACT scores are represented in green while lower ACT scores are represented in purple. Each category corresponds to around a quarter of students in our data set. Numbers above each data point refer to the number of responses from that group to that type of question and the error bars are 95% confidence intervals. Students tend to do better on non-CMC questions compared to CMC questions.

We then considered prior performance through the lens of standardized test scores. The result is shown in Fig 6 where the ACT score groups were chosen to correspond to roughly a quarter of students. We find that regardless of ACT score, students performed better on non-CMC questions. Notably, while performance on non-CMC questions tended to increase with ACT score, performance on CMC questions was largely unchanged.

Next, we examined performance on the CMC and non-CMC questions from the perspective of differentiating students within a course. That is, given a student who answered a CMC or non-CMC question correctly, what was the probability that the student would earn an “A” in Physics II? If CMC questions are better at discriminating between “A” students and other students, we would expect that answering CMC questions correctly would lead to a higher probability of earning an “A”. However, we find that there is no apparent relationship between students’ rate of accuracy on CMC and non-CMC questions and their probability of earning an “A” in Physics II (Table 2).

**Table 2:** Probability and 95% confidence intervals that a student who answered each type of multiple-choice question correctly would go on to earn an “A” in Physics II. We find that the probabilities are not statistically different from each other.

Type of Question	Probability	Total Number of Responses
CMC Questions	0.618 (0.584, 0.652)	1,301
Non-CMC Questions	0.602 (0.596, 0.609)	35,640

**Fig 7:**
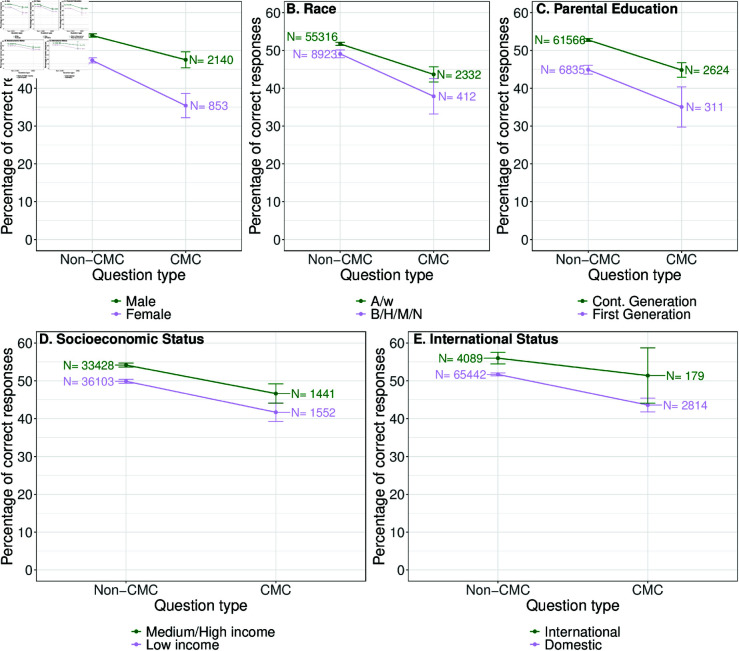
Comparison of the percentage of correct responses to CMC and non-CMC questions by Physics II students split by demographic groups. Error bars on each point represent 95% confidence intervals and the number above each point is the number of responses by that demographic group to that question type. We find all demographic groups perform worse on CMC questions than they perform on non-CMC questions.

We then looked at how various demographic groups performed on CMC and non-CMC questions (Fig 7). All groups performed better on the non-CMC questions, but the size of the performance gap varied between the groups. For example, the CMC-non-CMC performance gap was smallest for international students (4.6%) and largest for Black, Hispanic, Multiracial, and Native American students and female students (11% and 12% respectively). In general, we find that minoritized students have a larger performance gap between the two types of questions than their majoritized peers do, with the exception being first-generation status where the performance gap is 0.12 percentage points. The difference in the performance gap when minoritized students perform worse on CMC questions varies between 0.7% and 5.6%, with the smallest corresponding to the difference in performance gap between low-income and medium/high-income students and the largest corresponding to the difference in performance between male and female students. Practically, this result means that if male students on average had a performance penalty of δ percentage points on CMC questions compared to non-CMC questions, a female student on average would have a performance gap of δ+5.6 percentage points.

**Fig 8:**
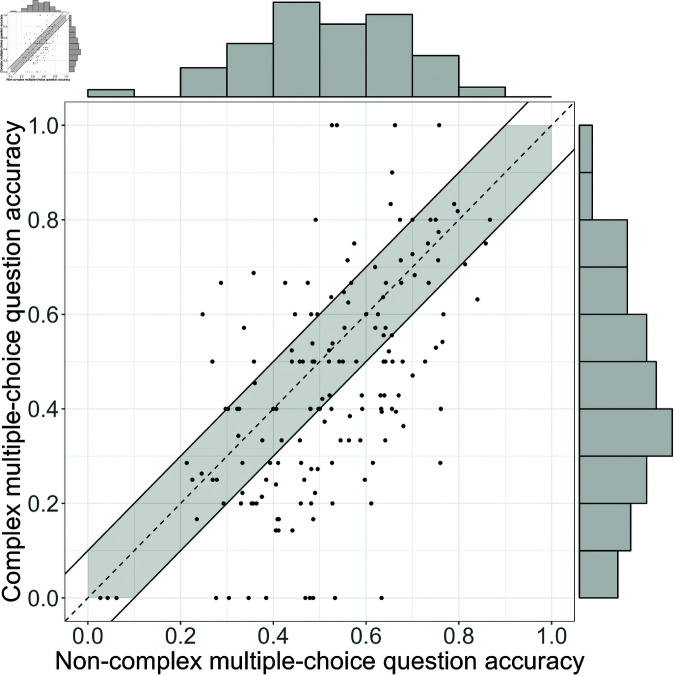
Comparison of student accuracy on non-CMC questions compared to CMC questions among students who answered at least five of each question type. The diagonal line denotes equal performance on the two types of questions. The gray region denotes where performance on the two types of questions are within ten percentage points of each other, or the equivalent of a letter grade on the standard scale where 90 to 100 equates to an A grade. The edge histograms show the relative number of students in each 10% band of accuracy. In general, students are less accurate at answering CMC than non-CMC questions, shown by there being more dots below the line of parity.

Finally, we compared individual student performance on the two types of questions (Fig 8). Of the students who saw at least five of each question type, most students (65.8%) answered a higher fraction of the non-CMC questions correctly compared to the CMC questions. Requiring a practically significant difference of at least ten percentage points, the equivalent of a full letter grade, we find that 44% of students performed better on the non-CMC questions, 15% performed better on the CMC questions, and 41% performed the same or within the 10 percentage point threshold (marked by the grey region in Fig 8).

For interested readers, additional plots with results split by demographics are included in S4 Appendix. Given limited sample sizes and that the results are not central to our argument, we do not discuss those results here.

### Regression models

To assess if those differences in performance gaps were significant, we developed regression models. As the bivariate models inform our interpretation of the multivariate model, we present those results first followed by the multivariate results. The bivariate results are shown in Table 3.

**Table 3:** Odds ratios for each regression model for answering a question correctly. *** *p* < .001, ** *p* < .01, and * *p* < .05. Numbers in parentheses represent 95% confidence intervals. The sex-question-type interaction is the only interaction found to be statistically significant.

Variable	Model 1	Model 2	Model 3	Model 4	Model 5	Model 6	Model 7
Intercept	0.856** (0.778, 0.941)	0.811*** (0.740, 0.890)	0.825*** (0.753, 0.904)	0.823*** (0.742, 0.912)	0.791*** (0.722, 0.860)	0.794*** (0.715, 0.881)	0.804*** (0.734, 0.882)
Question Type (CMC =1)	0.784 (0.600, 1.026)	0.753* (0.578, 0.980)	0.729* (0.559, 0.948)	0.680** (0.513, 0.901)	0.723* (0.556, 0.940)	0.727* (0.560, 0.945)	0.728* (0.560, 0.947)
Sex (female =1)	0.805*** (0.727, 0.892)						
Race (B/H/M/N=1)		0.906 (0.794, 1.035)					
Parental Education (First gen =1)			0.762*** (0.656, 0.884)				
Socioeconomic Status (Low income =1)				0.945 (0.859, 1.040)			
International Status (International = 1)					1.207 (0.978, 1.489)		
GPAO (centered at 3.492)						2.217*** (1.998, 2.459)	
ACT score (centered at 32.75 )							1.085*** (1.065, 1.105)
Sex * Question Type	0.813* (0.666, 0.992)						
Race * Question Type		0.820 (0.631, 1.067)					
Parental Education* Question Type			1.031 (0.758, 1.403)				
Socioeconomic Status * Question Type				1.140 (0.947, 1.371)			
International Status * Question Type					1.217 (0.844, 1.755)		
GPAO *Question Type						1.101 (0.874, 1.387)	
ACT Score * Question Type							0.983 (0.950, 1.018)

First, we notice that in most models, the question type variable is statistically significant and the odds ratio is less than one. This means that the reference group in each model (Asian and white students, continuing generation students, medium and high income students, domestic students) performs worse on CMC questions than traditional multiple choice questions.

Second, we notice that because the odds ratio is less than one for the sex and parental education variables, female and first-generation students perform worse on traditional multiple-choice questions than male and continuing-generation students do. Likewise, because the odds ratios are greater than one, we find that students with higher ACT scores and GPAOs perform better on traditional multiple choice questions than students with lower ACT scores and GPAOs do. For the other variables, (race, socioeconomic status, and international status) the odds ratio is not statistically different than one.

Finally, looking at the interaction terms, we notice that only the sex-question type interaction is statistically significant. Because the odds ratio for the interaction is less than one, the performance gap on CMC questions and traditional multiple choice questions is greater for female students than it is for male students.

To determine what the odds of a female student getting a CMC question correct are to the odds of the female student getting a non-CMC question correct, we can combine the odds ratios. For the coefficients, we would add them to get the simple effect so for odds ratios, we need to multiply them (because $e^{A+B}=e^{A}e^{B}$). For female students (sex=1), the odds ratio is the product of the question type odds ratio and the sex question type interaction odds ratio (0.784×0.813) and we find the odds of a female student answering a CMC question correctly are 0.637 the odds of a female student answering a non-CMC question correctly.

Based on the results of the bivariate models, model selection started from the null model (only question type) and then a single new variable and its interaction with question type were added one at a time, starting with sex followed by GPAO, ACT, parental education, international, race, and socioeconomic status. The log-likelihood and resulting p-value of the chi-squared test to compare each model with the previous are shown in Table 4. From the results, there is limited statistical evidence to add parental education or any of the subsequent variables to the model. Therefore, only the results of the multivariate model including question type, sex, ACT score, GPAO and their interactions with question type will be included here and are shown in Table 5. For interested readers, the odds ratios for the mixed effects model with all terms (model 7) are included in S5 Appendix.

**Table 4:** Model selection results. The Chi-squared statistic is calculated based on the difference in log likelihoods of the current row and previous row and the Δ degrees of freedom represents the change in the degrees of freedom between the current model and the model in the previous row. A p-value greater than 0.05 suggests the models are not statistically different. Based on the results, we selected model 3.

Model	Variables included in model	logLik	Chisq	Δ Df	p-value
Model 0	Question type	-39811			
Model 1	model 0 + sex and interaction	-39799	23.94	2	< 0.0001
Model 2	model 1 + GPAO and interaction	-39760	77.10	2	< 0.0001
Model 3	model 2 + ACT score and interaction	-39679	161.58	2	< 0.0001
Model 4	model 3 + parental education and interaction	-39679	0.12	2	0.9430
Model 5	model 4 + international and interaction	-39677	4.02	2	0.1341
Model 6	model 5 + race and interaction	-39675	3.35	2	0.1874
Model 7	model 6 + socioeconomic status and interaction	-39674	3.71	2	0.1562

**Table 5:** Odds ratios for mixed effects model with students and questions as crossed random effects. *** *p* < .001, ** *p* < .01, * *p* < .05, · *p* < .10. Numbers in parentheses represent 95% confidence intervals. The sex-question-type interaction was found to be marginally significant and all the other interactions were not statistically significant.

Variable	Odds Ratio
Intercept	0.851** (0.764, 0.949)
Question Type (CMC =1)	0.773· (0.591, 1.012)
Sex (female =1)	0.799*** (0.729, 0.876)
GPAO (centered at 3.492)	2.040*** (1.828, 2.276)
ACT (centered at 32.75)	1.039*** (1.020, 1.057)
Sex * Question Type	0.820· (0.672, 1.001)
GPAO * Question Type	1.183 (0.913, 1.532)
ACT * Question Type	0.974 (0.937, 1.013)

First, after controlling for sex and prior preparation, we find that question type has an odds ratio that is not statistically different from one, meaning that the odds of a male student with an average GPAO and ACT score answering a CMC question correctly are not statistically different than that of answering a non-CMC question correctly.

Second, we find that the demographic variable of sex has an odds ratio statistically different from one, with male students having higher odds of answering non-CMC questions correctly than female students do. We also, unsurprisingly, find that both measures of prior preparation have odds ratios statistically greater than one, meaning that higher grades in college courses and higher ACT scores are associated with increased accuracy on traditional (non-CMC) multiple choice questions.

Next, examining the interaction terms, we find mixed results. We find that the odds ratio of the sex-question type interaction is less than one, meaning the performance difference between male and female students is increased for CMC questions compared to non-CMC questions. Likewise, we find that the odds ratio of the interaction between ACT score and question type is less than one. In contrast, the odds ratio of the interaction between GPAO and question type is greater than one meaning that those with a higher GPAO have a smaller performance difference on CMC questions compared to non-CMC questions compared to those with lower GPAOs. However, we note that none of the interactions are statistically significant, though the sex question type interaction is marginally significant (p = 0.051).

Comparing the results of the multivariate model to the bivariate models, we find that the effects are in the same directions but whether they are statistically equivalent is not always the same. While question type and the sex question-type interaction were statistically significant in the bivariate models, they were not in the multivariate model. We examine possible reasons and explore this further in the discussion.

## Discussion

Here, we discuss each research question individually.

### How does students’ performance on CMC questions compare to their performance on non-CMC questions in introductory physics?

We found that students performed 7.9 percentage points worse on CMC questions compared to non-CMC questions. When looking at the results from the regression models, we found the odds ratio of question type to be statistically different from one in some of the models but not all of them. For example, the odds of a student with an average GPAO getting a CMC question correct is 0.727 of the odds of the student getting a non-CMC question correct. When the student’s ACT score and sex were added to the model, the effect was no longer statistically significant, meaning a male student with an average ACT score and GPAO has the same odds of getting a CMC question and a non-CMC question correct. Taken together, the results suggest that there is a performance gap on CMC questions compared to non-CMC questions though whether we can measure it depends on which group or sub-group of students we examine. Such results align with previous findings [12,18] that CMC questions tend to be more difficult than non-CMC questions.

### How does the type of knowledge students need to answer a question (conceptual vs. “plug-and-chug”) account for performance differences between CMC questions and non-CMC questions?

We did not find sufficient evidence that the type of knowledge students need to answer a question could account for the performance differences between CMC questions and non-CMC questions. We found that on non-CMC questions, student accuracy on “plug-and-chug” questions was 3.6 percentage points higher than it was on conceptual questions. As the 95% confidence intervals of the CMC/non-CMC performance difference and the “plug-and-chug” non-CMC/conceptual non-CMC performance differences do not overlap, these results suggest that the observed performance gap between CMC and non-CMC questions is not entirely due to CMC questions only being conceptual while the non-CMC questions were conceptual and “plug-and-chug”. In addition, that students performed worse on CMC questions than non-CMC conceptual questions further supports this conclusion.

### What is the relationship between students’ prior preparation and their ability to answer CMC questions correctly?

In terms of prior preparation, we find that “A” students answer CMC questions correctly at a higher rate than non-“A” students do. However, the difference in performance between CMC and non-CMC questions is around 8 percentage points, regardless of whether the student is an “A” student in their other courses or not.

From the regression models, we find that a higher GPAO is associated with increased odds of answering a non-CMC question correctly. However, the interaction term was not statistically different from one, suggesting that there is no disproportionate difference in performance on CMC and non-CMC questions based on the student’s GPAO.

We find a similar story with ACT scores. A higher ACT score was associated with increased odds of answering a non-CMC question correctly but we do not find an interaction effect. We believe this is likely because students with higher ACT scores do better on non-CMC questions than students with lower ACT scores, but everyone performs equally badly on CMC questions (Fig 6) and therefore, any performance difference between CMC and non-CMC questions is explained by differences in non-CMC question performance, which is what the ACT score variable in the model captures.

While we cannot address the potential for clueing directly, our results suggest that if clueing is occurring, it is not helping lower-ability students perform better on CMC questions by eliminating primary answer options due to the available secondary choices as hypothesized by Albanese [12]. If clueing were helping lower-ability students, we would expect the performance gap between CMC and non-CMC questions to be smaller for non-“A” students than for “A” students or for students with lower versus higher ACT scores because clueing would not help on the non-CMC questions. In addition, clueing would only benefit the non-“A” students and students with lower ACT scores and would be of little benefit to “A” students or students with higher ACT scores who were assumed to know the material. Instead, we found the performance gap to be similar.

We also note that performance on all questions is relatively low, regardless of the student’s prior preparation. For example, students who earned “A”s in physics II only answered around 60% of non-CMC questions correctly in our study. We discuss this more in the limitations.

### How well can a CMC question differentiate between students who go on to earn a high versus low grade in their physics course?

We find that CMC questions are able to differentiate between students who went on to earn “A”s in Physics II from those who did not. However, we do not find that CMC questions offer any practical differentiation benefits from non-CMC questions as the difference in performance gaps was 2 percentage points. We note that a recent paper found evidence that non-standard multiple-choice questions could be useful for differentiating high and low performing students [61] though that study did not include complex multiple choice questions. Therefore, while some non-standard multiple-choice formats may be useful for differentiating top-performing students from everyone else, our study does not support using CMC questions in this way.

When considering the probability that someone who answered a CMC question correctly would go on to earn an “A”, we find a similar result. While the probability that someone who answered a CMC question correctly earns an “A” in Physics II is higher by approximately 1.5 percentage points than the probability that someone who answered a non-CMC question correctly earns an “A” in Physics II, the result is not statistically or practically different.

### How might CMC questions affect performance gaps between students of various demographic groups?

We find that all demographic groups included in our study did worse on CMC than non-CMC questions. We also find that the differences between performance on CMC and non-CMC questions varied between demographic groups with international students having the smallest performance gap and Black, Hispanic, Multiracial, and Native American students and female students having the largest performance gaps. Prior work has also found evidence that there may be differential performance between male and female students on CMC questions [20].

From the regression models, we find mixed evidence that there may be an effect of CMC questions on performance based on demographics. We found a marginally significant interaction between sex and question type in our multivariate model after controlling for ACT score and GPAO and a significant interaction in our bivariate model. In addition, the values of the odds ratio of the interaction are similar in both the bivariate and multivariate models, which suggests that the interaction may hold explanatory power. If the interaction were instead a proxy, we would expect the value of the odds ratio to be much closer to one in the multivariate model because some of the effect would be “taken” by the other variables in the model.

It is possible however that the effect is only marginally significant because we are missing variables in our model and once those variables are included, we would find a different result [62]. Indeed, in multivariate model 7 (S5 Appendix), we do find the sex-question type interaction is significant once all demographic variables and their interactions are included in the model. However, our model selection process does not provide evidence that we would be justified picking the more complex model over the model we report in the paper. While some argue that it is justifiable to keep a non-statistically significant variable in the model provided there is a strong theoretical reason for doing so [55,62], we do not have a particularly strong theoretical reason for including the additional variables and our bivariate models do not provide strong experimental reasons for including additional variables beyond parental education. Including only parental education and its interaction with question type does not change the sex-question type interaction away from marginally significant. Therefore, additional evidence is needed to fully resolve this result. Regardless, it should be noted that whatever the result, it does not mean that the student’s sex causes a difference in performance but rather, that there is something different between male and female students in our data set that relates to students’ likelihood to answer the questions correctly.

For all other demographic groups, our regression models find limited evidence that CMC questions could exacerbate existing performance gaps.

We note that the question type variable in the models is confounded by the fact that non-CMC questions are a mix of conceptual and “plug-and-chug” questions while CMC questions are only conceptual and the conclusions should be interpreted through that lens.

## Limitations and future work

Our study has a few limitations that might affect the generalizability and applicability of our results. Here, we describe them as well as potential ways to address them in future studies.

First, our study did not occur in an actual testing environment and hence, the results might not necessarily hold there. Students may respond to questions differently based on if the questions are completed in a practice versus high-stakes examination format. That “A” earning students did not answer Problem Roulette questions with the accuracy that would be expected of them (e.g., >80% on average) suggests that this is the case. We would therefore expect increased performance on questions if this study were repeated in a high-stakes environment.

Future work can then examine CMC questions in high-stakes environments, such as class exams, rather than the low-stakes environment examined here to address this first limitation. The increased stress and time pressure of taking an exam could have an additional performance effect not seen in a low-stakes environment. Alternatively, there could be an effect working in the opposite direction in that students are more careful and thorough when answering exam questions compared to practice, not-for-credit questions.

Future work could also examine the potential usefulness of CMC questions in learning environments rather than only assessment contexts. For example, perhaps the increased difficulty of CMC questions could result in increased learning relative to non-CMC questions if these questions are used as part of pre-class quizzes or in-class clicker questions and students are encouraged to reflect on their incorrect answers.

Second, our study did not directly compare similar CMC and non-CMC questions; that is, question content could be a confounding variable. Instead, our results are based on comparing overall performance on CMC and non-CMC questions and only partially separates out any effect based on whether the question is a conceptual question or a “plug-and-chug” question. Our random effects approach largely takes care of potential content differences from a modeling perspective, but it does not take into account the type of content tested. Future work could then compare performance on as similar as possible CMC and non-CMC questions to minimize the impact of confounding factors like question content.

At the same time, CMC questions are only one variant of alternatives to traditional multiple-choice questions; therefore, future work could examine the affordances and limitations that each provides in the context of physics. For example, multiple-response multiple-choice questions have been previously used in physics as an alternative to free-response questions [63,64] but work in other fields suggests this type of question is even more difficult than CMC questions for students [65]. Alternatively, multiple-true-false questions strike a balance between the benefits of multiple responses while not being as difficult as CMC questions [65]. Other studies suggest that students may actually prefer multiple-true-false questions compared to traditional multiple-choice questions [66,67].

Third, this study was conducted at an institution where the student population is a particular subset of the overall physics-taking population [59]. As such, we should not necessarily assume that the results would be applicable to institutions with greater representations of Black, Hispanic, Multi-racial, and Native American students or low-income students, for example. To ensure that these studies are broadly applicable, future research should be conducted at a variety of institutions under various conditions to simulate how students might engage with the material.

In addition, the data set used in this study is based on students who opted in to the Problem Roulette service, which could be a confounding factor. A comparison of students who used Problem Roulette compared to students who did not is included in the supplemental material (S1 Appendix) and suggests that differences between students who did and did not opt-in to Problem Roulette are likely minimal.

Finally, future work should examine CMC questions in disciplines other than physics. As introductory physics is considered relatively difficult [68], it is possible that the CMC penalty observed here might be underestimated for other disciplines where students did not find the material as difficult (or, alternatively, where low grades overall are not as common).

## Conclusions and recommendations

We found students performed worse on CMC than non-CMC questions. We found this to be true regardless of the students’ prior preparation and course grade, their demographics, and the type of knowledge needed to answer the question. From the lens of differentiating top students from other students, we do not recommend using CMC questions for this purpose based on our results.

We did find possible evidence that CMC questions may disproportionately harm female students more than male students. Using CMC questions in a physics assessment could then be an equity issue in the sense that some students might be penalized more than others. Additional work should be conducted to substantiate this effect and ensure the results translate to high-stakes testing environments rather than just the low-stakes environment studied here.

Finally, while other studies recommend against CMC questions more broadly, our study does not have a wide enough scope to make such a global recommendation. For example, our study does not allow us to determine whether the increased difficulty of CMC questions aids in learning compared to traditional multiple choice questions. We do recommend that if instructors choose to include CMC questions or other non-standard multiple choice questions on their assessments, students should be given ample opportunity to practice answering those types of questions beforehand. Otherwise, the use of non-standard question formats such as CMC could potentially create inequities simply because some students are familiar with the question format while others are not.

## Supporting information

S1 Appendix Comparison of students who used Problem Roulette to those who did not. In this appendix, we compare the demographics, grades, and ACT scores of those who used the optional Problem Roulette service from those who did not
(PDF)

S2 Appendix Terms to identify CMC questions. In this appendix, we list the terms we used to identify possible CMC questions.
(PDF)

S3 Appendix Examples of conceptual and “plug-and-chug” questions. In this appendix, we provide three examples of conceptual questions and three examples of “plug-and-chug” questions.
(PDF)

S4 Appendix Additional Question Accuracy Plots. In this appendix, we provide plots of the CMC vs non-CMC accuracy plots (Fig 8) split by demographics.
(PDF)

S5 Appendix Regression results (model 7). In this appendix, we provide the regression results from the full model, referred to as model 7 in Table 4.
(PDF)
