# Supplementary material for: Exploring how complex multiple-choice questions could contribute to inequity in introductory physics
Source: PLoS One. 2025 May 30;20(5):e0323813. doi: 10.1371/journal.pone.0323813 (PMC12124580; doi:10.1371/journal.pone.0323813)
Supplement: S1 Appendix — In this appendix, we compare the demographics, grades, and ACT scores of those who used the optional Problem Roulette service from those who did not (PDF) [file pone.0323813.s001.pdf]

Exploring how complex multiple-choice questions could contribute to inequity in introductory physics

Comparison of students who used Problem Roulette to those who did not

Here, we compare Problem Roulette users in Physics II to everyone who took Physics II during the study period to provide additional context to our results.

First, we compared the grades students typically earned in their other classes for both Problem Roulette and non-Problem Roulette users (Fig. 1). We find that overall, the distributions are similar; however, the median student who used Problem Roulette had a slightly higher GPAO compared to students in the course overall.

Second, we compared the ACT scores of students in the two groups (Fig. 2). Again, we notice that the distributions are statistically similar for both groups of students.

Third, we compared the grades students earned in Physics II (Fig. 3) and find that students who earned "A"s and "B"s are slightly overrepresented in the Problem Roulette data compared to everyone who took Physics II at our university.

Finally, we looked at the demographic distributions of students who used Problem Roulette compared to the overall course (Fig. 4). We find that for most groups, the students using Problem Roulette are representative of students in the overall course. Sex is the exception to this as female students are slightly overrepresented among Problem Roulette users in this sample.

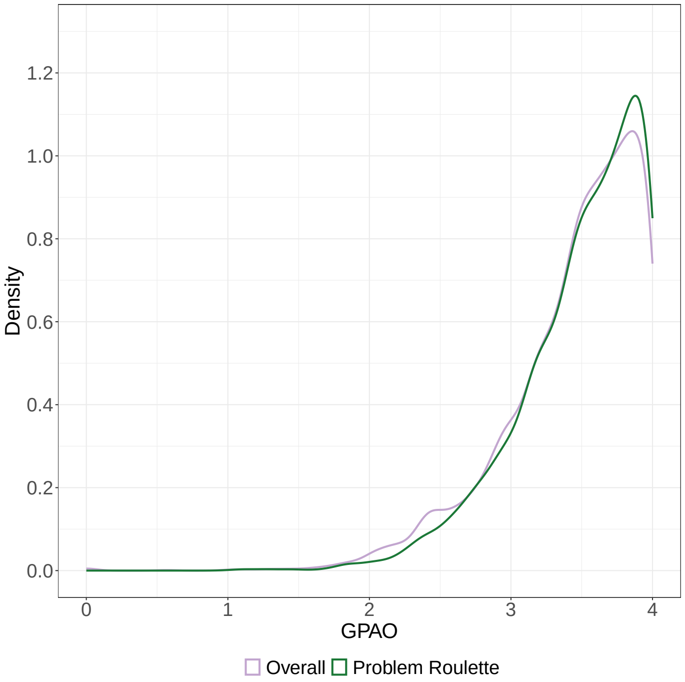

Figure 1: Comparison of the distributions of students who earned each grade (on average) in their other courses for students who used our Problem Roulette system and for all students in the course.

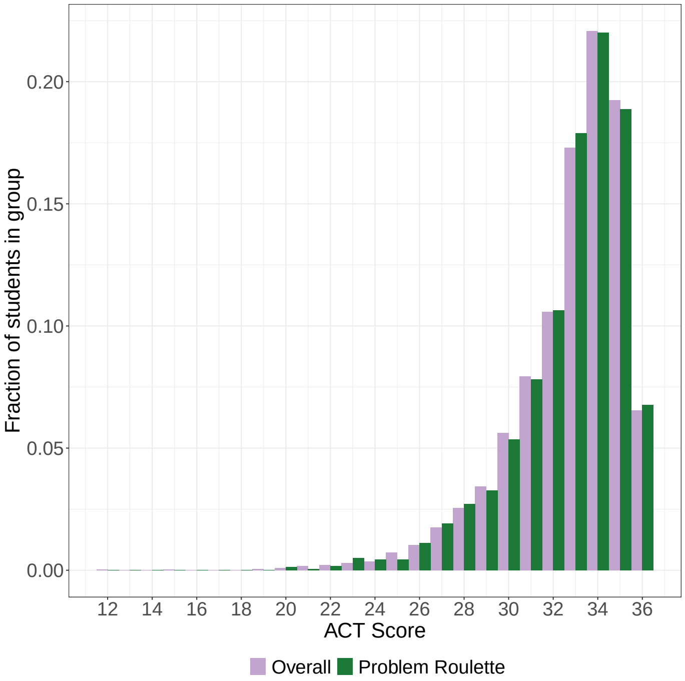

Figure 2: Comparison of the distribution of students who earned each ACT score for students who used our Problem Roulette system and for all students in the course.

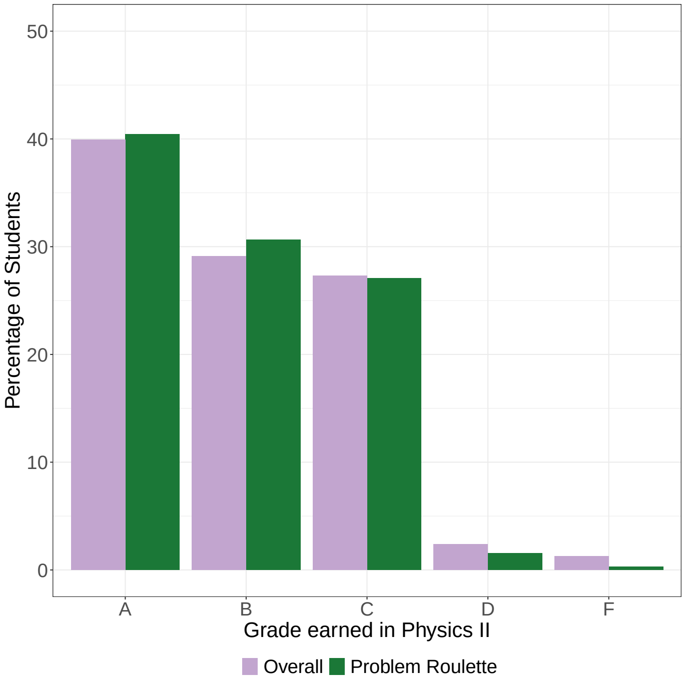

Figure 3: Comparison of the percentage of students who earned each grade in Physics II for students who used our Problem Roulette system and for all students in the course.

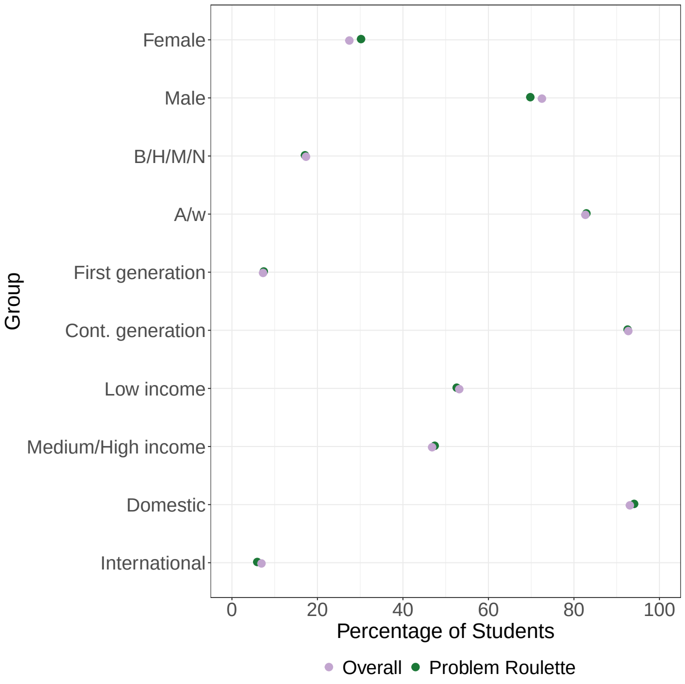

Figure 4: Comparison of the percentage of students based on demographics for students who used our Problem Roulette system and for all students in the course.
