# Supplementary material for: Exploring how complex multiple-choice questions could contribute to inequity in introductory physics
Source: PLoS One. 2025 May 30;20(5):e0323813. doi: 10.1371/journal.pone.0323813 (PMC12124580; doi:10.1371/journal.pone.0323813)
Supplement: S2 Appendix — In this appendix, we list the terms we used to identify possible CMC questions. (PDF) [file pone.0323813.s002.pdf]

Exploring how complex multiple-choice questions could contribute to inequity in introductory physics

Terms to identify CMC questions

Here, we list the terms we searched for when identifying CMC questions: “a and b”, “a and c”, “a and d”, “a and e”, “b and c”, “b and d”, “b and e”, “c and d”, “c and e”, “d and e”, “a & b only”, “a & c only”, ”a & d only”, “a & e only”, “b & c only”, “b & d only”, “b & e only”, “c & d only”, “c & e only”, ”d & e only”, ”a, b, and c”, “a, b, and d”, “a, b, and e”, “b, c, and d”, “b, c, and e”, ”c, d, and e”, ”both a and b”, “both a and c”, “both a and d”, “both a and e”, “both b and c”, ”both b and d”, ”both b and e”, ”both c and d”, “both c and e”, “both d and e”, “(i) only”, “(ii) only”, “(iii) only”, “(i) and (ii)”, “(i) and (iii)”, ”(ii) and (iii)”, “(i), (ii), and (iii)”, ”(i) and (ii) only”, “(i) and (iii) only”, “i and ii”, “i and iii”, “i and iv”, “ii and iv”, “ii and iii”, “iii and iv”, “i, ii, and iii”, “i, ii, and iv”, “ii, iii, and iv”, “1 only”, “2 only”, “3 only”, “1 and 2 only”, “1 and 3 only”, “1 and 4 only”, “2 and 4 only”, “3 and 4 only”, “1 and 2”, “1 and 3”, “1 and 4”, “1 and 5”, “2 and 3”, “2 and 4”, “2 and 5”, “3 and 4”, “3 and 5”, “1, 2, and 3”, “statements 1, 2, 3, and 5 only.”, “statements 2 and 5 only.”, “statements 2, 3, 4, and 5 only.”, “statements 2, 3, and 5 only.”, “statements 3 and 5 only.”, “1, 2, and 3 only”, “1, 2, and 4 only”, “1, 2, and 5 only”, “1, 3, and 4 only”, “1, 3, and 5 only”, “1, 4, and 5 only”, “2, 3, and 4 only”, “2, 3, and 5 only”, “2, 4, and 5 only”, “3, 4, and 5 only”, “either b) or c)”, “any of a), b), or c)”, “all of a), b), and c)”, “both a) and b)”, “both b) and c)”, “both a) and c)”, “either a) or b)”, “either a) or c)”, “one statement”, “two statements”, “three statements”, “all four statements”, “four statements”.
