# Supplementary material for: Exploring how complex multiple-choice questions could contribute to inequity in introductory physics
Source: PLoS One. 2025 May 30;20(5):e0323813. doi: 10.1371/journal.pone.0323813 (PMC12124580; doi:10.1371/journal.pone.0323813)
Supplement: S3 Appendix — In this appendix, we provide three examples of conceptual questions and three examples of “plug-and-chug” questions. (PDF) [file pone.0323813.s003.pdf]

Exploring how complex multiple-choice questions could contribute to inequity in introductory physics

Examples of conceptual and "plug-and-chug" questions

Here, we provide examples of non-CMC questions from Problem Roulette as well as whether we classified them as conceptual or "plug-and-chug" questions. The correct option is in bold.

Conceptual examples

1. A positively charged particle is placed at rest on one of the electric field lines in the above diagram and then released. Which of the following statements most accurately describes its motion?

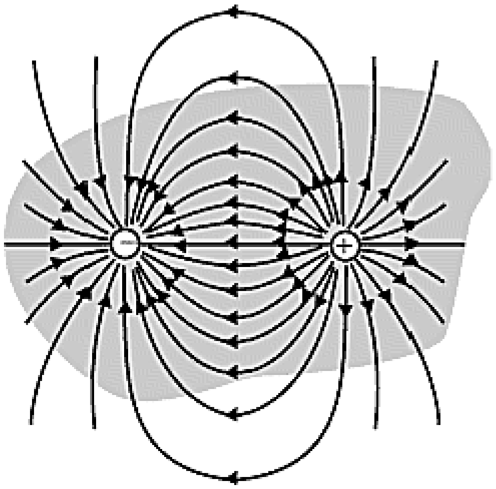

A. Its acceleration will always be tangent to a field line.
B. It will move perpendicular to the field lines at a constant velocity.
C. It will accelerate perpendicular to the field lines.
D. It will move along the field line at a non-constant velocity.
E. It will move at a constant velocity along the field line.

2. What is the y component of the electric field, E_y, at point P a distance R out on the perpendicular from one end of a uniformly negatively charged rod of length L and charge -Q? (take Q to be a positive quantity)

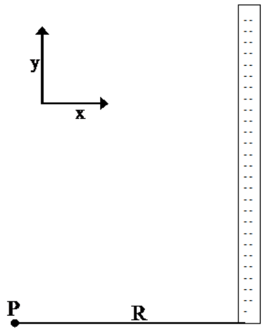

A. $\frac{-kQ}{L}\int_0^L \frac{y\,dy}{(y^2+R^2)}$

B. $\frac{kQ}{L}\int\limits_0^L \frac{y\,dy}{(y^2+R^2)^{3/2}}$

C. $\frac{kQ}{L}\int_0^L \frac{dx}{(x^2+R^2)^{3/2}}$

D. $\frac{kQ}{L}\int_0^L \frac{R\,dy}{(y^2+R^2)}$

E. $\frac{kQ}{L}\int_0^L \frac{R\,dy}{(y^2+R^2)^{3/2}}$

3. A metal ring is dropped from rest below a bar magnet that is fixed in position as suggested in the figure. An observer views the ring from below. Which one of the following statements concerning this situation is true?

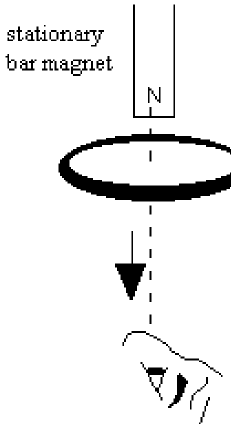

A. As the ring falls, there will be an induced magnetic field around the ring that appears clockwise as viewed by the observer.

B. As the ring falls, there will be an induced magnetic field around the ring that appears counterclockwise as viewed by the observer.

C. Since the magnet is stationary, there will be no induced current in the ring.

D. As the ring falls, an induced current will flow clockwise as viewed by the observer.

E. As the ring falls, an induced current will flow counterclockwise as viewed by the observer.

"Plug-and-chug" examples

1. Four point charges are placed at the corners of a square as shown in the figure. Each side of the square has length 2.0 m. Determine the magnitude of the electric field at the point P, the center of the square.

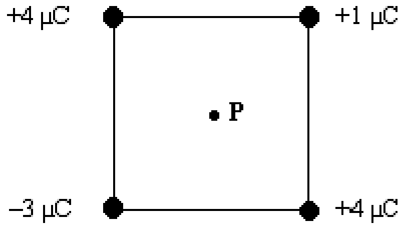

A. 3×10^{-6} N/C

B. 1.8×10^{-6} N/C

C. 2.7×10^{-4} N/C

D. 9.0×10^{-3} N/C

E. 2.0×10^{-6} N/C

2. In the circuit shown below, the voltage across the 0.5 mF (m=10-3) capacitor when the circuit is in its steady state is 7 Volts. Now the polarity of the 8V battery is reversed (i.e. it is changed so that the positive terminal is above the negative terminal in the diagram). Immediately after this happens, what is the current through the capacitor? (Assume that the battery terminals are reversed quickly enough that the charge and voltage on the capacitor do not change during that process)

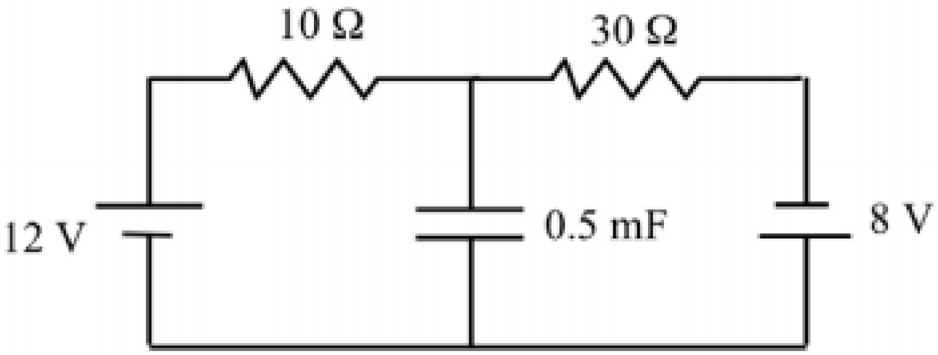

A. 0.22 Amps
B. 0.03 Amps
C. 0 Amps
D. 0.53 Amps
E. 0.85 Amps

3. An electric dipole is held at rest so its dipole moment of 0.2 C-m makes an angle of 40 degrees with a uniform electric field of 130 N/C. If we release the dipole so it is free to turn without friction with its moment of inertia of I = 15 kg m^2, what is the dipole's maximum angular velocity, ω, in radians/second?

A. 8.2
B. 0.9
C. 0.35
D. 0.052
E. 2.7
