# Supplementary material for: Exploring how complex multiple-choice questions could contribute to inequity in introductory physics
Source: PLoS One. 2025 May 30;20(5):e0323813. doi: 10.1371/journal.pone.0323813 (PMC12124580; doi:10.1371/journal.pone.0323813)
Supplement: S4 Appendix — In this appendix, we provide plots of the CMC vs non-CMC accuracy plots (Fig 8) split by demographics. (PDF) [file pone.0323813.s004.pdf]

Exploring how complex multiple-choice questions could contribute to inequity in introductory physics

Additional Question Accuracy Plots

Here, we include additional plots to provide context for our data.

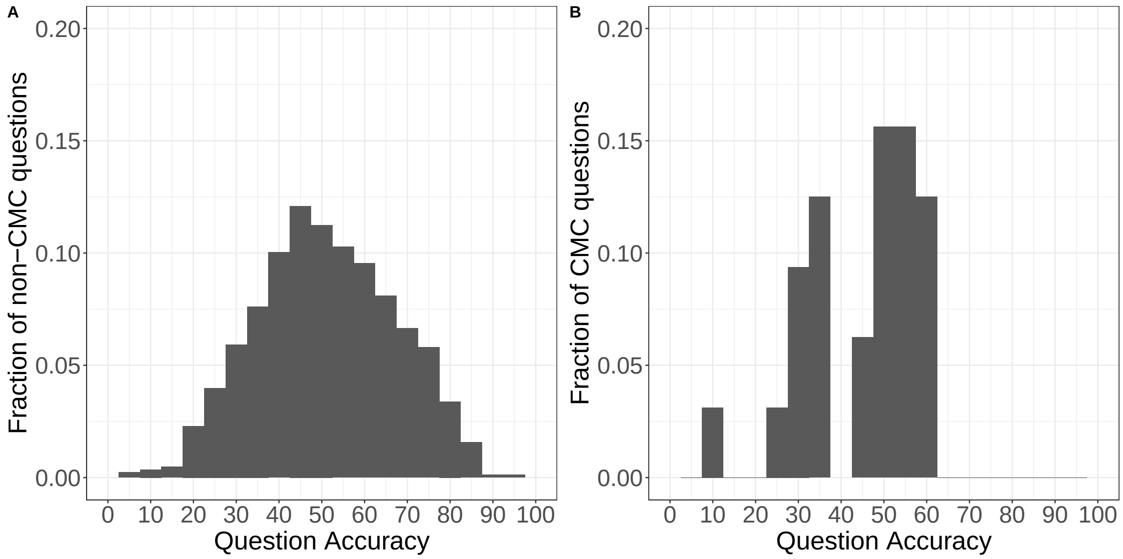

Figure 1: The distribution of accuracy to the individual non-CMC (A) and CMC (B) questions. Questions without at least 10 responses are not included in the plot. The bin width is set to 5 percentage points. While the non-CMC questions have a bell-like distribution, the CMC questions do not. No CMC question had more than 65% of the responses marked as correct.

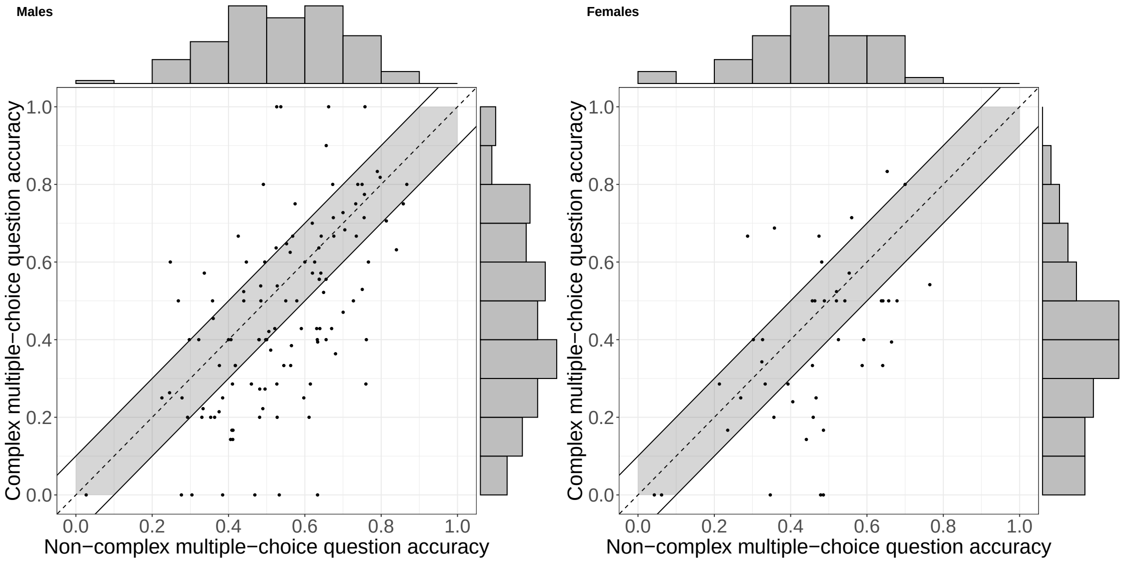

Figure 2: Comparison of student accuracy on non-CMC compared to CMC questions among students who answered at least five of each question type split by the student's sex. The diagonal line denotes equal performance on the two types of questions. The gray region denotes where performance on the two types of questions are within ten percentage points of each other.

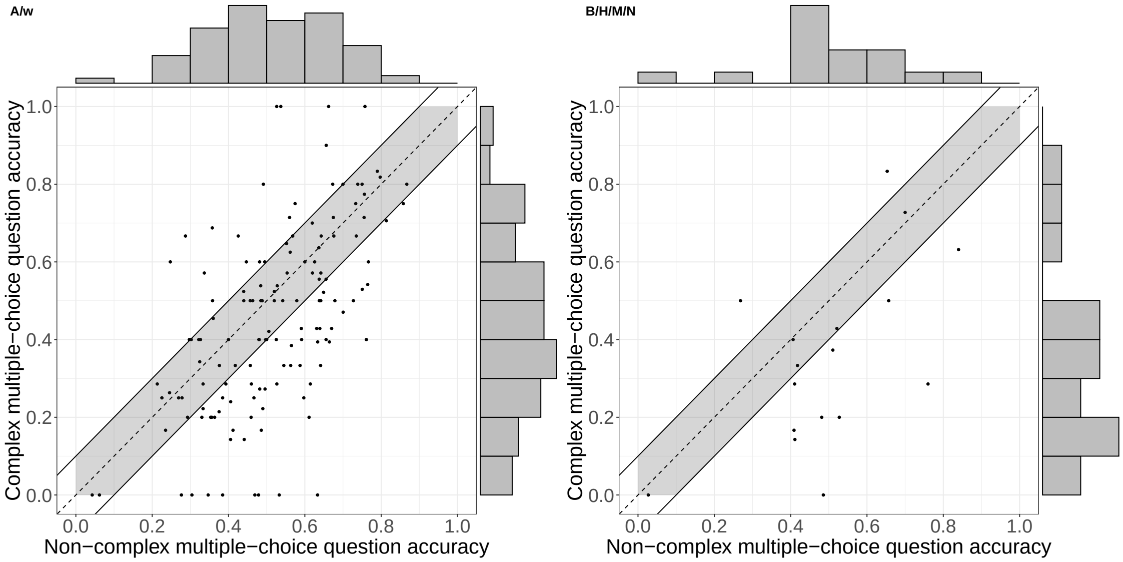

Figure 3: Comparison of student accuracy on non-CMC compared to CMC questions among students who answered at least five of each question type split by the student's race. The diagonal line denotes equal performance on the two types of questions. The gray region denotes where performance on the two types of questions are within ten percentage points of each other.

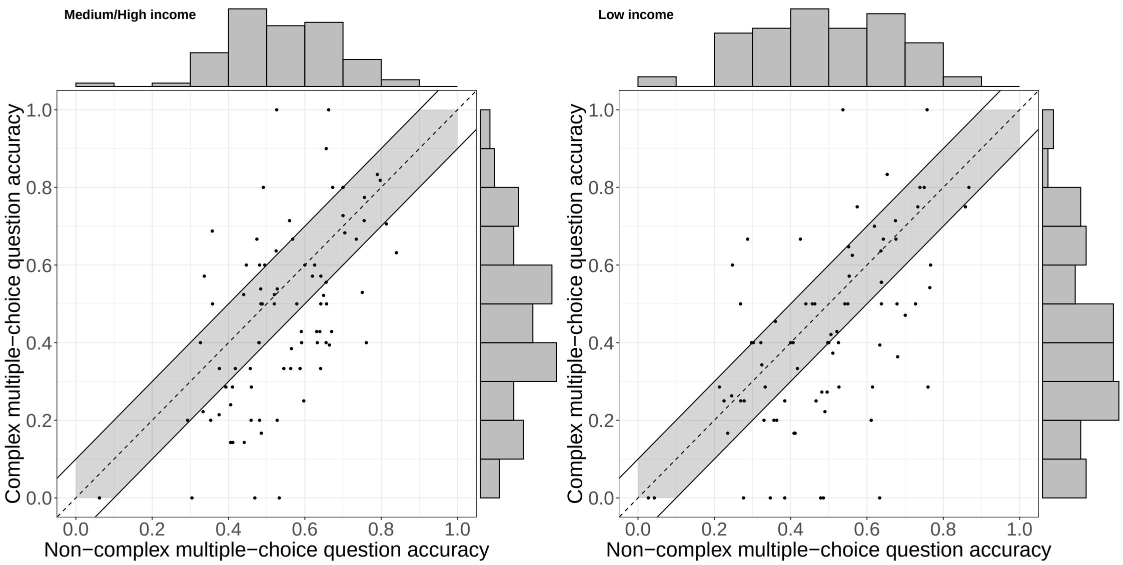

Figure 4: Comparison of student accuracy on non-CMC compared to CMC questions among students who answered at least five of each question type split by the student's socioeconomic status. The diagonal line denotes equal performance on the two types of questions. The gray region denotes where performance on the two types of questions are within ten percentage points of each other.

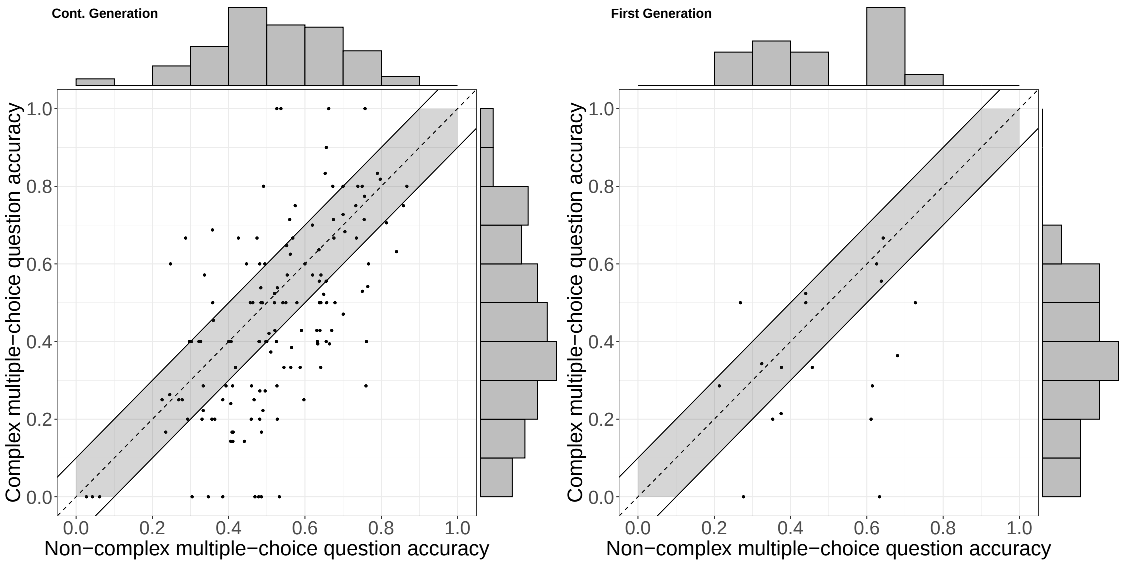

Figure 5: Comparison of student accuracy on non-CMC compared to CMC questions among students who answered at least five of each question type split by the student's parent's highest education level. The diagonal line denotes equal performance on the two types of questions. The gray region denotes where performance on the two types of questions are within ten percentage points of each other.

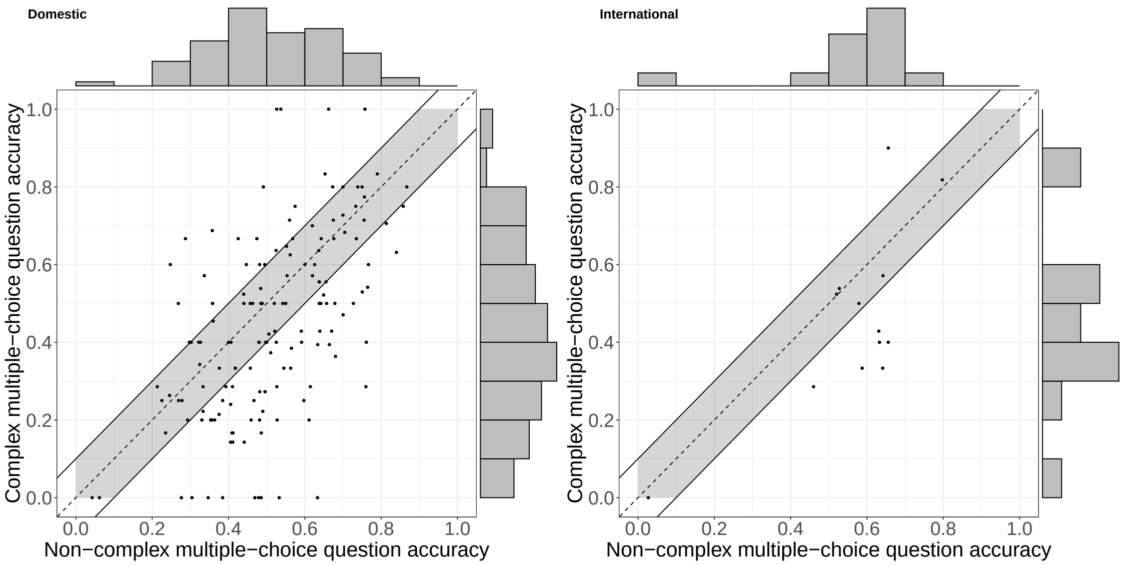

Figure 6: Comparison of student accuracy on non-CMC compared to CMC questions among students who answered at least five of each question type split by the student's residency. The diagonal line denotes equal performance on the two types of questions. The gray region denotes where performance on the two types of questions are within ten percentage points of each other.
