# Supplementary material for: Exploring how complex multiple-choice questions could contribute to inequity in introductory physics
Source: PLoS One. 2025 May 30;20(5):e0323813. doi: 10.1371/journal.pone.0323813 (PMC12124580; doi:10.1371/journal.pone.0323813)
Supplement: S5 Appendix — In this appendix, we provide the regression results from the full model, referred to as model 7 in Table 4. (PDF) [file pone.0323813.s005.pdf]

Exploring how complex multiple-choice questions could contribute to inequity in introductory physics

Regression results (model 7)

Here, we present the results of the full mixed model before we performed any model selection. The model presented in the paper (model 3) is the one selected from the model selection procedure. This model is only included for completeness.

Table 1: Odds ratios for mixed effects model with students and questions as crossed random effects; *** $p < .001$, ** $p < .01$, * $p < .05$, · $p < .10$.

Variable	Odds Ratio
Intercept	0.864* (0.765, 0.976)
Question Type (CMC =1)	0.716* (0.532, 0.963)
Sex (female =1)	0.800*** (0.730, 0.877)
Race (B/H/M/N=1)	1.035 (0.917, 1.167)
Parental Education (First gen =1)	1.017 (0.884, 1.170)
Socioeconomic Status (Low income =1)	0.962 (0.882, 1.049)
International Status (International = 1)	1.126 (0.931, 1.361)
GPAO (centered at 3.492)	2.042*** (1.828, 2.282)
ACT (centered at 32.75)	1.039*** (1.020, 1.059)
Sex * Question Type	0.811* (0.667, 1987)
Race * Question Type	0.763· (0.576, 1.011)
Parental Education * Question Type	1.036 (0.754, 1.422)
Socioeconomic Status * Question Type	1.191· (0.986, 1.437)
International Status * Question Type	1.343 (0.924, 1.951)
GPAO * Question Type	1.198 (0.923, 1.555)
ACT * Question Type	0.964· (0.926, 1.002)
